# Rapamycin Inhibits IGF-1 Stimulated Cell Motility through PP2A Pathway

**DOI:** 10.1371/journal.pone.0010578

**Published:** 2010-05-11

**Authors:** Lei Liu, Long Chen, Yan Luo, Wenxing Chen, Hongyu Zhou, Baoshan Xu, Xiuzhen Han, Tao Shen, Shile Huang

**Affiliations:** 1 Department of Biochemistry and Molecular Biology, Louisiana State University Health Sciences Center, Shreveport, Louisiana, United States of America; 2 Feist-Weiller Cancer Center, Louisiana State University Health Sciences Center, Shreveport, Louisiana, United States of America; University of Minnesota, United States of America

## Abstract

Serine/threonine (Ser/Thr) protein phosphatase 2A (PP2A) has been implicated as a novel component of the mammalian target of rapamycin (mTOR) signaling pathway. Recently we have demonstrated that mTOR regulates cell motility in part through p70 S6 kinase 1 (S6K1) and eukaryotic initiation factor 4E (eIF4E) binding protein 1 (4E-BP1) pathways. Little is known about the role of PP2A in the mTOR-mediated cell motility. Here we show that rapamycin inhibited the basal or insulin-like growth factor 1 (IGF-1)-induced motility of human Ewing sarcoma (Rh1) and rhabdomyosarcoma (Rh30) cells. Treatment of the cells with rapamycin activated PP2A activity, and concurrently inhibited IGF-1 stimulated phosphorylation of Erk1/2. Inhibition of Erk1/2 with PD98059 did not significantly affect the basal mobility of the cells, but dramatically inhibited IGF-1-induced cell motility. Furthermore, inhibition of PP2A with okadaic acid significantly attenuated the inhibitory effect of rapamycin on IGF-1-stimulated phosphorylation of Erk1/2 as well as cell motility. Consistently, expression of dominant negative PP2A conferred resistance to IGF-1-stimulated phosphorylation of Erk1/2 and cell motility. Expression of constitutively active MKK1 also attenuated rapamycin inhibition of IGF-1-stimulated phosphorylation of Erk1/2 and cell motility. The results suggest that rapamycin inhibits cell motility, in part by targeting PP2A-Erk1/2 pathway.

## Introduction

The mammalian target of rapamycin (mTOR), a member of the phosphoinositide-3′ kinase-related kinase family, is a central controller of cell proliferation, growth and survival [1]. Rapamycin can form a complex with FK506 binding protein 12 (FKBP-12) and then bind mTOR, selectively inhibiting its kinase activity and function [1]. Recently, two mTOR complexes (mTORC1 and mTORC2) have been identified in mammalian cells [1]. mTORC1 is composed of mTOR, mLST8 (also termed G-protein β-subunit-like protein, GβL, a yeast homolog of LST8), PRAS40 (proline-rich Akt substrate 40 kDa) and raptor (regulatory-associated protein of mTOR), and is rapamycin-sensitive [2]–[8]. In response to growth factors and nutrients, mTORC1 regulates cell proliferation and growth by modulating many processes, including protein synthesis and ribosome biogenesis through downstream effectors like 4E-BP1 (eukaryotic initiation factor 4E binding protein 1) and S6K1 (ribosomal p70 S6 kinase 1) [1]. mTORC2 consists of mTOR, mLST8, mSin1 (mammalian stress-activated protein kinase-interacting protein 1), rictor (rapamycin insensitive companion of mTOR), and PRR5 (proline-rich protein 5), and is rapamycin-insensitive [9]–[15]. mTORC2 phosphorylates Akt [12], [14], [16], and controls cytoskeleton organization and cell survival [10], [11]. Most recently, mTORC2 has been reported to phosphorylate SGK1 (serum and glucocorticoid-inducible kinase 1) [17], although this remains controversial [18]. Both mTORC1 and mTORC2 interact with a negative regulator DEPTOR [19].

Clinical trials have demonstrated that rapamycin and its analogs (CCI-779, RAD001 and AP23573) (termed rapalogs) are promising anticancer drugs. They share same mechanism and specifically block the function of mTOR, inhibiting growth of numerous solid tumors (renal, breast, prostate, colon and brain cancers) with only mild side effects [20]. Intensive studies have focused on the crucial roles of mTOR in controlling cell proliferation, growth and survival. Recently this laboratory and others have further revealed its pivotal role in regulating cell migration [21]–[26]. We found that rapamycin suppresses IGF-1 stimulated F-actin reorganization and migration in various tumor cell lines by inhibiting mTORC1-mediated 4E-BP1 and S6K1 pathways [23]. This is in part associated with rapamycin inhibition of phosphorylation of the focal adhesion proteins (FAK, paxillin and p130^Cas^) [24].

PP2A, a serine/threonine (Ser/Thr) protein phosphatase, is a heterotrimeric holoenzyme composed of a catalytic subunit (PP2Ac), an A subunit (also termed PR65), and members of the B subunit families, such as B (PR55), B′ (PR61), B″ (PR72), and B′″ (PR93/PR110) [27]. The phosphatase activity of PP2Ac is modulated by its association with PP2A-A, and -B regulatory subunits [27]. Of interest, PP2A has been identified as the phosphatase responsible for the dephosphorylation of S6K1 and 4E-BP1, and inhibition of mTOR with rapamycin has been shown to stimulate these PP2A-mediated events [28], suggesting that PP2A is a novel downstream target of mTOR. It has been suggested that α4 protein, the mammalian homolog of yeast Tap42 [29], associates with PP2Ac [30]. Association of α4 with PP2A, PP4, and PP6 has been linked to rapamycin sensitivity [30]–[32]. In yeast TOR phosphorylates Tap42 and promotes its association with Pph21/22 and Sit4 (encoding PP2Ac and PP6, respectively), inhibiting their activities [29], [33], although there exists disputation [34], [35]. As in yeast, the consequence of α4 dissociation from PP2A or PP6 in mammalian cells is contradictory. For instance, rapamycin has been reported to inhibit cell proliferation by decreasing PP2A activity through dissociation α4 from the PP2Ac [36]. However, other studies [31], [34] do not demonstrate rapamycin-induced dissociation of α4 from PP2A or PP6.

PP2A negatively regulates Erk1/2 [37], [38], though this notion is controversial [39]. Activation of Erk1/2 by IGF-1 is associated with mitogenesis and cell motility [40]. Inhibition of PP2A activity promotes motility in a number of transformed cells and cancer cell lines [41], whereas activation of PP2A by β_2_-adrenergic receptor agonists inhibits motility of keratinocytes [38]. Because PP2A is negatively regulated by mTOR, we hypothesized that mTOR may regulate cell motility in part through PP2A-Erk1/2 pathway. This study was designed to test the hypothesis.

## Materials and Methods

### Cell culture

Human rhabdomyosarcoma Rh30 and Ewing sarcoma Rh1 (gifts from Dr. Peter J. Houghton, St Jude Children's Research Hospital, Memphis, TN, USA) were grown in antibiotic-free RPMI 1640 medium (Mediatech, Herndon, VA, USA) supplemented with 10% fetal bovine serum (FBS) (Hyclone, Logan, UT, USA) at 37°C and 5% CO_2_. Human embryonic kidney (HEK) 293 (American Type Culture Collection, Manassas, VA), 293TD and 293A cells (Invitrogen, Carlsbad, CA, USA) were grown in antibiotic-free Dulbecco's modified Eagle medium (DMEM) (Mediatech) supplemented with 10% heat-inactivated FBS and non-essential amino acid (Mediatech) at 37°C and 5% CO_2_. For experiments where cells were deprived of serum, cell monolayers were washed with phosphate buffered saline (PBS), and incubated in the serum-free DMEM. Rh1 and Rh30 clones [42] stably expressing empty vector pcDNA3 and AU1-tagged rapamycin-resistant mutant mTOR (S2035I), mTORrr, were generously provided by Dr. Peter J. Houghton (St. Jude Children's Research Hospital, Memphis, TN). Rh1 and Rh30 clones stably expressing hemagglutinin (HA)-tagged dominant-negative (dn) protein phosphatase 2A catalytic subunit (PP2Ac) (L199P) (dn-PP2A) were described previously [43]. The above clones were expanded in growth medium containing 500 µg/ml G418 for further experiments. Individual clones expressing mTORrr and dn-PP2A were examined by Western blotting with antibodies to AU1 and HA, respectively. The functions of the mutant proteins in the cells were further confirmed by detection of phosphorylation of S6K1 and 4E-BP1 (for mTORrr) and Erk1/2 (for dn-PPP2A), as described [42], [43].

### Recombinant adenoviral constructs and infection of cells

The recombinant adenovirus expressing the green fluorescence protein (GFP) (Ad-GFP) was described previously [23]. To construct recombinant adenoviruses expressing FLAG-tagged constitutively active and dominant negative MKK1, DNA fragments encoding the corresponding mutants were excised from pMCL-MKK1-R4F and pMCL-MKK1-K97M [44] (gifts from Dr. Natalie Ahn, University of Colorado, Boulder, CO, USA), and then sub-cloned to FLAG-tagged pENTR11 shuttle vector. The recombinant adenovirus was generated using ViraPower™ Adenoviral Gateway™ Expression Kit (Invitrogen, Carlsbad, CA, USA) following the manufacture's instruction. All adenoviruses were amplified, titrated and used as described [23], [24].

### Lentiviral shRNA cloning, production, and infection

Lentiviral shRNAs to mTOR and GFP were described previously [24]. For use, monolayer cells, when grown to about 70% confluence, were infected with above lentiviral shRNAs in the presence of 8 µg/ml polybrene for 12 h twice at an interval of 6 h. Uninfected cells were eliminated by exposure to 2 µg/ml puromycin for 48 h before use.

### Western blot analysis

Western blotting was performed as described previously [23]. The primary antibodies used included antibodies to FLAG, β-tubulin (Sigma, St. Louis, MO, USA), HA, Erk2, MKK1, mTOR (FRAP), p-S6K1 (Thr389), S6K1 (Santa Cruz Biotechnology, Santa Cruz, CA, USA), and p-Erk1/2 (Thr202/Tyr204) (Cell Signaling, Beverly, MA, USA).

### *In vitro* PP2A phosphatase assay

Cells were lysed in 50 mM Tris-HCl buffer, pH 7.0, containing 1% Nonidet P-40, 2 mM EDTA, and protease inhibitor cocktail (Sigma, 1∶1000). PP2Ac was immunoprecipitated with antibodies to PP2Ac (Millipore, Temecula, CA, USA), and protein A/G-agarose (Santa Cruz Biotechnology). Subsequently, the beads were washed three times with the above lysis buffer, and twice with the phosphatase assay buffer (50 mM Tris-HCl, pH 7.0, 0.1 mM CaCl_2_). The phosphatase activity of immunoprecipitated PP2A was assayed with a Ser/Thr Phosphatase Assay kit 1 using KRpTIRR as the substrate peptide (Millipore) following the manufacturer's instructions. Absorbance was measured at 595 nm using a Wallac 1420 Multilabel Counter (PerkinElmer Life Sciences, Wellesley, MA, USA). Because the optical density (OD) values varied from experiment to experiment, PP2A activity was calculated using the fold change (arbitrary units) in each experiment. Finally all data (from different batches of experiments) were pooled for statistical analysis.

### Cell motility assay

Cell motility was determined using the wound healing assay, as described previously [23].

### Statistical analysis

Results were expressed as mean values ± standard error (mean ± SE). The data were analyzed by one-way analysis of variance (ANOVA) followed by post-hoc Dunnett's *t*-test for multiple comparisons. A level of *P*<0.05 was considered to be significant.

## Results

### Rapamycin activates protein phosphatase 2A in an mTOR-dependent manner

**Figure 1:**
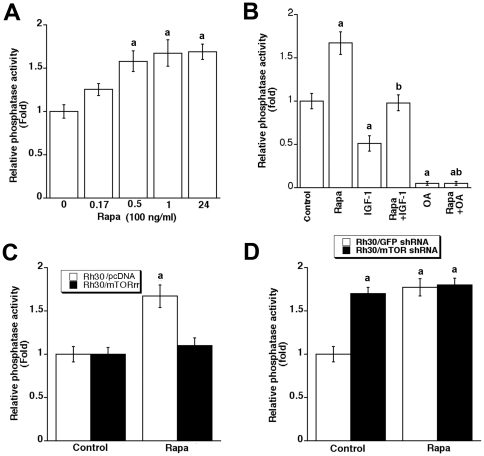
Rapamycin activates PP2A in an mTOR-dependent manner. Serum-starved Rh30 cells were exposed to rapamycin (Rapa, 100 ng/ml) for the indicated time (A), or pretreated with or without rapamycin (Rapa, 100 ng/ml) or okadaic acid (OA, 100 nM) for 2 h, and then stimulated with or without IGF-1 (10 ng/ml) for 1 h (B), or the indicated serum-starved Rh30 cells were exposed to rapamycin (Rapa, 100 ng/ml) for 2 h (C, D). PP2A in cell lysates was immunoprecipitated with antibodies to PP2A catalytic subunit (PP2Ac) plus protein A/G agarose beads, followed by *in vitro* phosphatase assay using Ser/Thr Phosphatase Assay Kit 1 (Millipore). Data represent mean ± SE from 3–4 independent experiments. ^a^ *P*<0.05 *vs.* controls, ^b^ *P*<0.05 *vs.* Rapa group.

Our previous studies have shown that rapamycin suppresses IGF-1 stimulated motility in various tumor cell lines in part by inhibiting mTORC1-mediated 4E-BP1 and S6K1 pathways [23]. Since PP2A, a negative regulator of cell motility [41], has been implicated as a novel downstream target of mTOR [28], we determined whether rapamycin inhibits cell motility by activating PP2A. We found that under serum-free conditions, exposure to rapamycin for 30 min resulted in a significant increase of the phosphatase activity of PP2A in Rh30 cells (Fig. 1A). The rapamycin-induced activation of PP2A was sustained for at least 24 h (Fig. 1A). Furthermore, we found that IGF-1 decreased the activity of PP2A, which was prevented by rapamycin (Fig. 1B). As expected, okadaic acid (OA), an inhibitor of PP2A, inhibited the basal PP2A activity by ∼90%, and also completely blocked rapamycin-induced PP2A activity (Fig. 1B).

To understand whether rapamycin activates PP2A through inhibition of mTOR, Rh1 and Rh30 cells stably expressing a rapamycin-resistant mutant mTOR (S2035I), mTORrr, that prevents binding of FKBP-rapamycin but has intact kinase activity, were used. We found that expression of mTORrr did not significantly reduce the basal PP2A activity, but conferred high resistance to rapamycin inhibition of PP2A activity in Rh30 cells (Fig. 1C). Similar data were observed in Rh1/mTORrr cells (data not shown), indicating that rapamycin-induced activation of PP2A is mTOR-dependent. This is further supported by our findings that downregulation of mTOR by ∼85% with lentiviral shRNA increased the PP2A activity by approximately 1.8 fold, mimicking the effect of rapamycin on PP2A (Fig. 1D). Our results support the notion that PP2A lies downstream of mTOR and is negatively regulated by mTOR.

### Rapamycin suppresses IGF-1 stimulated phosphorylation of Erk1/2 in an mTOR-dependent manner

**Figure 2:**
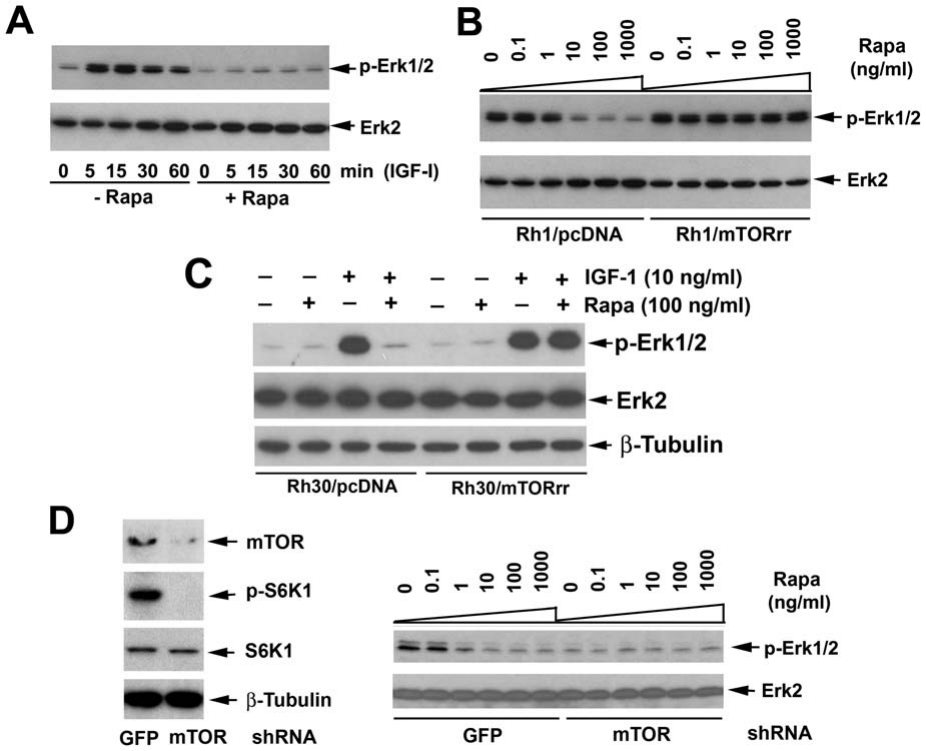
Rapamycin suppresses IGF-1 stimulated phosphorylation of Erk1/2 in an mTOR-dependent manner. (A) Serum starved Rh1 cells were pretreated with or without rapamycin (Rapa, 100 ng/ml) for 2 h, and then stimulated with IGF-1 (10 ng/ml) for the indicated time. (B) Serum-starved Rh1/pcDNA and Rh1/mTORrr cells were exposed to increasing concentrations of rapamycin (Rapa, 0–1000 ng/ml) for 2 h, and then to IGF-1 for 15 min. (C) Serum-starved Rh30/pcDNA and Rh30/mTORrr cells were pretreated with or without rapamycin (Rapa, 100 ng/ml) for 2 h, and then stimulated with or without IGF-1 (10 ng/ml) for 15 min. (D) Serum-starved Rh1 cells, infected with lentiviral shRNA to GFP (control) or mTOR, were stimulated with IGF-1 (10 ng/ml) for 1 h (Left panel), or exposed to increasing concentrations of rapamycin (Rapa, 0–1000 ng/ml) for 2 h, and then to IGF-1 for 15 min. For (A–D), cell lysates were analyzed by Western blotting using indicated antibodies.

Since Erk1/2, negatively regulated by PP2A [37], [38], have been implicated in the regulation of cell motility [39], we next investigated whether the inhibitory effect of rapamycin on cell motility is through downregulation of Erk1/2. We found that treatment of cells with IGF-1 (10 ng/ml) or rapamycin (100 ng/ml) for ∼2 h did not significantly alter the protein levels of Erk1/2 (Fig. 2A). However, IGF-1 induced robust activation of Erk1/2, as detected by Western blotting with an antibody that recognizes phospho-Erk1/2 (Thr202/Tyr204). Activation of Erk1/2 was rapid but transient. Phosphorylation was maximal after 5 min of IGF-1 stimulation, and then declined. Of interest is that IGF-1-induced activation of Erk1/2 was dramatically blocked by rapamycin, at concentrations of 10–1,000 ng/ml, in parental Rh1 (Fig. 2A) or Rh30 cells (data not shown).

To unveil whether rapamycin inhibits phosphorylation of Erk1/2 through inhibition of mTOR, Rh1 and Rh30 cells stably expressing mTORrr were used. As shown in Fig. 2B and 2C, expression of mTORrr did not obviously alter the basal or IGF-1-induced phosphorylation of Erk1/2, but conferred high resistance to rapamycin inhibition of IGF-1-induced phosphorylation of Erk1/2 in Rh1 and Rh30 cells, suggesting an mTOR-dependent mechanism involved. This is further supported by the following RNA interference studies. Consistent with our previous findings [24], lentiviral shRNA to mTOR silenced expression of mTOR protein by ∼85% in Rh30 cells, comparing with the control shRNA (to GFP). Downregulation of mTOR, mimicking the effect of rapamycin, dramatically decreased the mTOR kinase activity, since the IGF-1-stimulated phosphorylation of S6K1 (T389), routinely used as an indicator of mTOR kinase activity [1], was not detectable by Western blotting (Left panel, Fig. 2D). As expected, knockdown of mTOR, but not GFP, abrogated IGF-1-induced phosphorylation of Erk1/2 in Rh1 cells (Right panel, Fig. 2D). Similar results were also observed in mTOR-downregulated Rh30 cells (data not shown).

### Inhibition of PP2A with okadaic acid confers resistance to rapamycin inhibition of IGF-1-induced phosphorylation of Erk1/2 and cell motility

**Figure 3:**
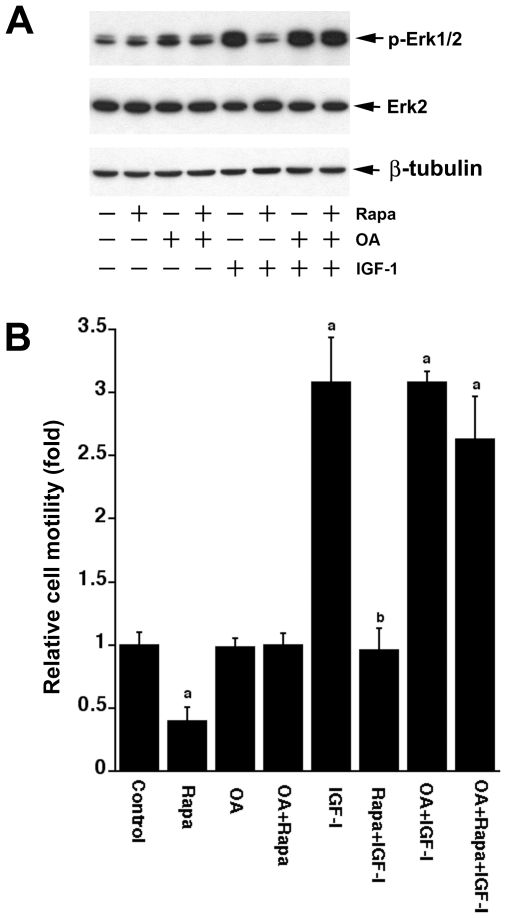
Inhibition of PP2A with okadaic acid confers resistance to rapamycin inhibition of IGF-1-induced phosphorylation of Erk1/2 and cell motility. (A) Serum-starved Rh1 cells were pretreated treated with or without OA (100 nM) for 1 h, and then treated with or without rapamycin (Rapa, 100 ng/ml) for 2 h, followed by stimulation with or without IGF-1 (10 ng/ml) for 15 min. Cell lysates were analyzed by Western blotting using indicated antibodies. β-tubulin served as loading control. (B) Motility of Rh1 cells was determined by the wound healing assay, as described in *Materials and Methods*. Following wounding, serum-starved Rh1 cells were pretreated treated with or without OA (100 nM) for 1 h, and then treated with or without rapamycin (Rapa, 100 ng/ml) for 2 h, followed by stimulation with or without IGF-1 (10 ng/ml) for 22 h. Cells migrated over the denuded area were observed and photographed with an Olympus inverted phase-contrast microscope equipped with Quick Imaging system. The number of cells migrating per millimeter of scratch was counted. Results are means ± SE of 3–4 independent experiments. ^a^ *P*<0.05, difference *vs.* control group; ^b^ *P*<0.05, difference *vs.* IGF-1 group.

It has been described that insulin or IGF-1 stimulates activation of S6K1, which is attributed to mTOR repression of PP2A activity against S6K1 [28]. To determine whether a similar mechanism could explain rapamycin inhibition of IGF-1 activation of Erk1/2, we used okadaic acid (OA), a relatively specific PP2A inhibitor [45]. Because okadaic acid selectively inhibits PP2A without inhibiting PP1 in intact cells at concentrations up to 100 nM [45], serum-starved Rh1 cells were treated with rapamycin (100 ng/ml) in the presence of OA (100 nM). As shown in Fig. 3A, IGF-1 (10 ng/ml, 5 min) stimulated Erk1/2 phosphorylation, and this effect was inhibited by pretreatment with rapamycin (100 ng/ml, 2 h). Pretreatment with OA for 1 h reversed the inhibitory effect of rapamycin on IGF-1-induced phosphorylation of Erk1/2. OA also slightly increased the basal level of phosphorylation of Erk1/2 in the absence or presence of rapamycin (Fig. 3A). Similar results were obtained with calyculin A, another PP2A inhibitor (data not shown). These results support the findings that a cross-talk occurs between mTOR and MAP kinases pathways [46]. IGF-1 stimulation of mTOR represses PP2A, resulting in activation of Erk1/2 and this is prevented by rapamycin.

Next, we investigated the role of PP2A in mTOR-mediated cell motility. To this end, a monolayer of Rh1 or Rh30 cells were grown in 6-well plates to 80% confluence, and serum-starved in DMEM for 24 h. Cell motility was assessed by the wound healing assay, as described previously [23]. Following wounding, cells were pretreated with or without OA (100 nM) for 1 h, then treated with or without rapamycin (100 ng/ml) for 2 h, followed by stimulation with or without IGF-1 (10 ng/ml) for 22 h. Consistent with our previous findings [23], [24], rapamycin inhibited the basal or IGF-1-stimulated cell motility by 60–70% in Rh1 cells (Fig. 3B). IGF-1 stimulated the cell motility by ∼3-fold, and this was dramatically attenuated by rapamycin to the basal level (Fig. 3B). OA alone did not affect the basal or IGF-1 stimulated cell motility significantly, but conferred high resistance to rapamycin inhibition of the basal or IGF-1-stimulated cell motility in Rh1 cells (Fig. 3B). Similar results were also observed in Rh30 cells (data not shown).

### Expression of dominant negative PP2A confers resistance to rapamycin inhibition of cell motility

**Figure 4:**
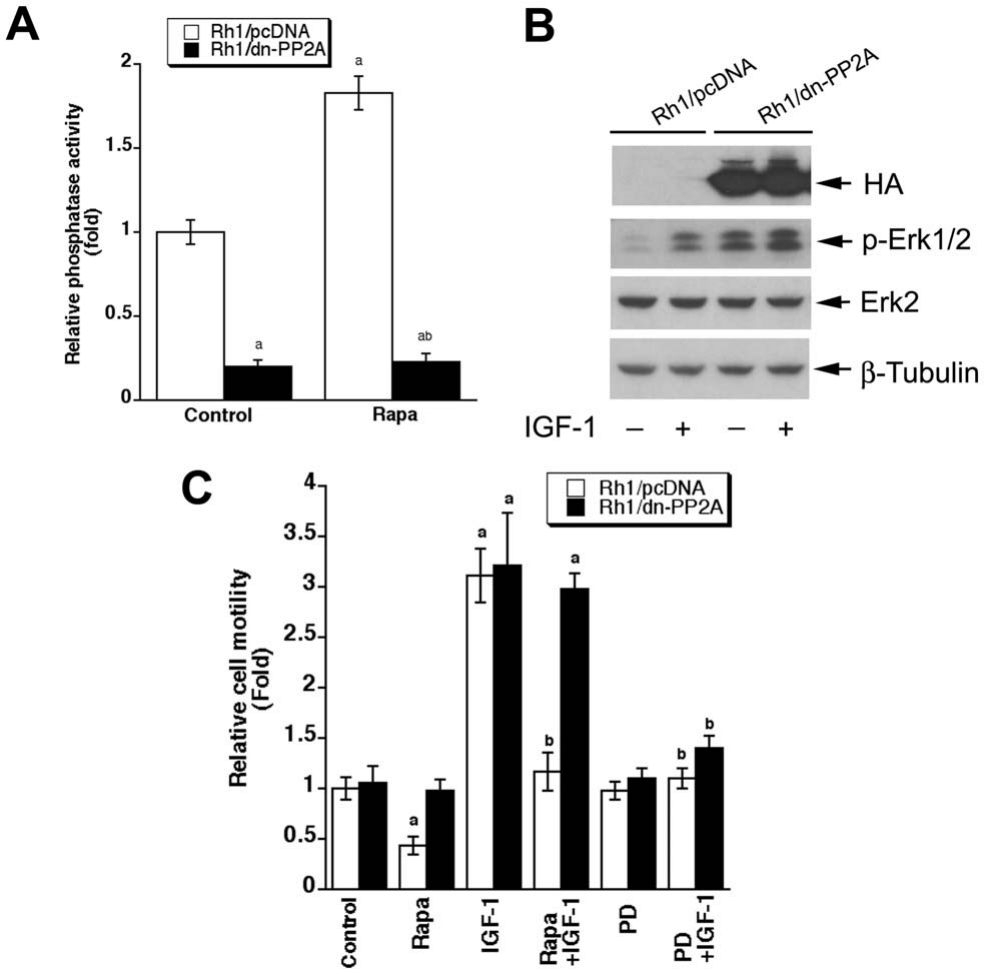
Expression of dominant negative PP2A confers resistance to rapamycin inhibition of cell motility. (A) Rh1 cells were stably transfected with vector alone (Rh1/pcDNA) or with a vector expressing HA-tagged dn-PP2Ac (L199P), serum-starved for 24 h, and then treated with or without rapamycin (Rapa, 100 ng/ml), followed by *in vitro* phosphatase assay using Ser/Thr Phosphatase Assay Kit 1 (Millipore). Data represent mean ± SE from 3–4 independent experiments. ^a^ *P*<0.05 *vs.* Rh1/pcDNA control, ^b^ *P*<0.05 *vs.* Rh1/pcDNA Rapa group. (B) Serum-starved Rh1/pcDNA and Rh1/dn-PP2A cells were stimulated with or without IGF-1 (10 ng/ml) for 15 min, followed by Western blotting using the indicated antibodies. (C) Motility of Rh1/pcDNA and Rh1/dn-PP2A cells was determined by the wound healing assay, as described in *Materials and Methods*. Serum-starved cells were pretreated with or without rapamycin (Rapa, 100 ng/ml) or PD98059 (PD, 10 µM) for 2 h, and then stimulated with or without IGF-1 (10 ng/ml) for 22 h. Results are means ± SE of 3–4 independent experiments. ^a^ *P*<0.05, difference *vs.* control group; ^b^ *P*<0.05, difference *vs.* IGF-1 group.

To further determine the role of PP2A in the regulation of Erk1/2 activity and cell motility, Rh1 and Rh30 cells were engineered to stably express HA-tagged dominant negative PP2A (L199P, dn-PP2A). Consistent with previous findings [47], expression of dn-PP2A, but not an empty vector (pcDNA3), reduced the phosphatase activity of PP2A by approximately 80% in serum-starved Rh1 cells (Fig. 4A). Rapamycin was able to activate PP2A in the control cells, but not in the cells expressing dn-PP2A (Fig. 4A). When the cells were serum-starved for 24 h, and then stimulated with or without IGF-1 (10 ng/ml) for 10 min, a low basal level of phosphorylation of Erk1/2 (Thr202/Tyr204) was observed in Rh1/pcDNA cells (control), whereas considerable level of phospho-Erk1/2 could be detected in Rh1/dn-PP2A cells. IGF-1 was able to stimulate phosphorylation of Erk1/2 in Rh1/pcDNA and Rh1/dn-PP2A cells (Fig. 4B). Also, due to failure to activate PP2A in the cells expressing dn-PP2A (Fig. 4A), rapamycin did not obviously inhibit IGF-1-induced phospho-Erk1/2 in Rh1/dn-PP2A cells either (data not shown). Similar results were observed in Rh30/pcDNA and Rh30/dn-PP2A cells (data not shown). Of interest, expression of dn-PP2A did not significantly affect the basal cell motility, but conferred high resistance to rapamycin inhibition of the basal or IGF-1 stimulated cell motility (Fig. 4C). Furthermore, we also noticed that cells expressing dn-PP2A remained sensitive to an Erk1/2 inhibitor, PD98059 (Fig. 4C).

### Inhibition of Erk1/2 blocks IGF-1-induced cell motility

**Figure 5:**
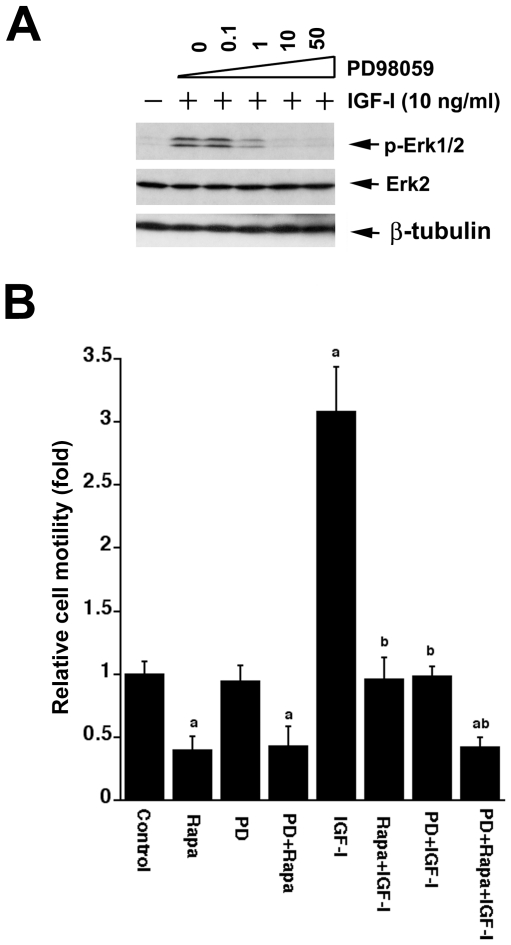
Inhibition of Erk1/2 with PD98059 blocks IGF-1-stimulated cell motility. (A) Serum-starved Rh1 cells were exposed to increasing concentrations of PD98059 (0–50 µM) for 2 h, and then to IGF-1 for 15 min, followed by Western blotting using the indicated antibodies. (B) Motility of Rh1 cells was determined by the wound healing assay. Serum-starved cells were pretreated for 2 h with rapamycin (Rapa) (100 ng/ml) or PD98059 (PD) (10 µM) alone, or in combination as indicated, followed by stimulation with or without IGF-1 (10 ng/ml) for 22 h. Results are means ± SE of 3–4 independent experiments. ^a^ *P*<0.05, difference *vs.* control group; ^b^ *P*<0.05, difference *vs.* IGF-1 group.

To address whether activation of Erk1/2 by IGF-1 is responsible for increased cell motility, cells were exposed to PD98059 (a selective inhibitor of MKK1/2, upstream of Erk1/2) alone, or in combination with rapamycin. We found that PD98059 did not alter the basal level of Erk1/2 phosphorylation (data not shown), but at concentrations of 1–50 µM, significantly inhibited IGF-1-induced activation of Erk1/2 in Rh1 cells (Fig. 5A). PD98059 (10 µM) alone did not significantly affect the basal mobility of cells, but obviously inhibited IGF-1-induced cell motility (Fig. 5B). However, in combination with rapamycin, PD98059 did not show a significant additive or synergistic effect on rapamycin inhibition of IGF-1-stimulated cell motility (Fig. 5B). Similar results were obtained with U0126 (5 µM), another MKK1/2 inhibitor (data not shown).

### Expression of constitutively active MKK1 confers resistance to rapamycin inhibition of cell motility

**Figure 6:**
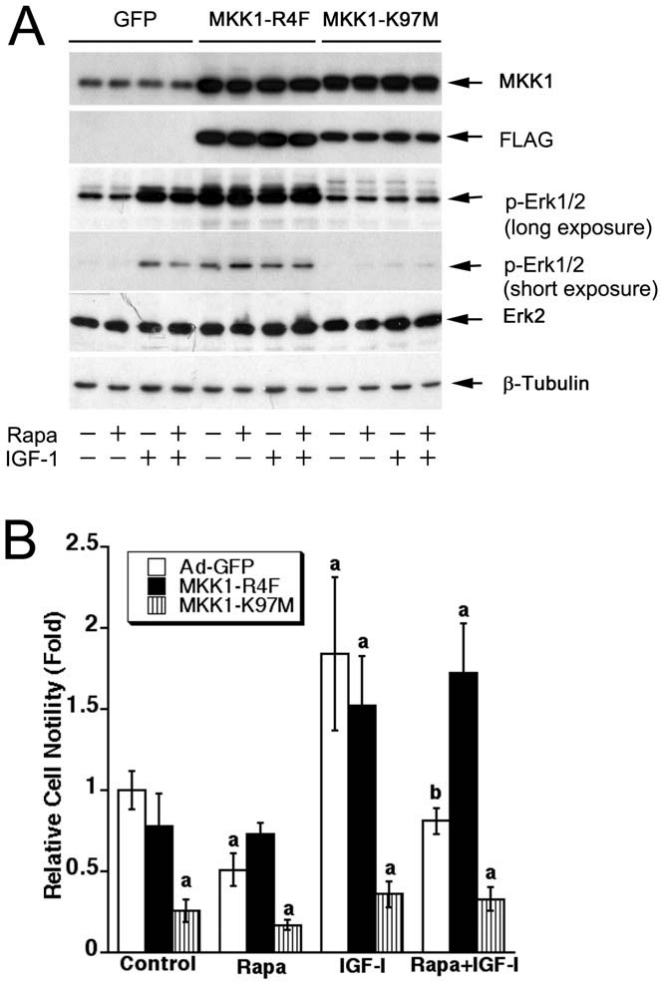
Expression of constitutively active MKK1 confers resistance to rapamycin inhibition of cell motility. (A) Rh30 cells, infected with Ad-MKK1-R4F, Ad-MKK1-K97M, and Ad-GFP (control), respectively, were serum-starved for 24 h, pretreated with or without rapamycin (Rapa, 100 ng/ml) for 2 h, and then stimulated with or without IGF-1 (10 ng/ml) for 15 min, followed by Western blotting with indicated antibodies. (B) Motility of Rh30 cells infected with Ad-MKK1-R4F, Ad-MKK1-K97M or Ad-GFP was determined by the wound healing assay. Results are means ± SE of 3–4 independent experiments. ^a^ *P*<0.05, difference *vs.* Ad-GFP control group; ^b^ *P*<0.05, difference *vs.* IGF-1 group.

To further define the role of Erk1/2 in mTOR-mediated cell motility, we generated recombinant adenoviruses Ad-MKK1-R4F and Ad-MKK1-K97M, encoding FLAG-tagged constitutively active and dominant negative MKK1, respectively. Infection of Rh30 cells with Ad-MKK1-R4F and Ad-MKK1-K97M, but not Ad-GFP (control virus), resulted in expression of high levels of FLAG-tagged MKK1 mutants (Fig. 6A). Expression of MKK1-R4F resulted in robust phosphorylation of Erk1/2 even without stimulation with IGF-1, whereas expression of MKK1-K97M blocked IGF-1-stimulated phosphorylation of Erk1/2 (Fig. 6A), indicating that the MKK1 mutants function as expected. Of interest, expression of MKK1-R4F, but not GFP or MKK1-K97M, in Rh30 cells prevented rapamycin inhibition of IGF-1 stimulated phosphorylation of Erk1/2 (Fig. 6A). Expression of MKK1-R4F slightly reduced the basal or IGF-1 stimulated cell motility, but conferred high resistance to rapamycin inhibition of the basal or IGF-1-induced cell motility (Fig. 6B). In contrast, expression of MKK1-K97M in the cells significantly inhibited the basal or IGF-1 stimulated cell motility (Fig. 6B).

## Discussion

Recently we have demonstrated that rapamycin inhibits F-action reorganization and cell motility by inhibition of mTOR-mediated S6K1 and 4E-BP1/eIF4E pathways [23], [24]. Since PP2A, a negative regulator of Erk1/2 and cell motility [37]–[39], has been implicated as a novel downstream target of mTOR [28], this study was set out to determine the role of PP2A-Erk1/2 pathway in mTOR-mediated cell motility. Here we show that treatment with rapamycin activated PP2A and concurrently suppressed IGF-1 stimulated phosphorylation of Erk1/2 in human Ewing sarcoma (Rh1) and rhabdomyosarcoma (Rh30) cells. Inhibition of PP2A with OA, or expression of dominant negative PP2A significantly attenuated the inhibitory effect of rapamycin on IGF-1-stimulated phosphorylation of Erk1/2 as well as cell motility. Furthermore, expression of constitutively active MKK1 resulted in robust phosphorylation of Erk1/2 and also attenuated rapamycin inhibition of IGF-1-stimulated cell motility. The results suggest that rapamycin inhibits cell motility, in part by targeting PP2A-Erk1/2 pathway.

The phosphatase activity of PP2Ac is modulated by its association with PP2A-A, -B regulatory subunits and α4 protein [27]. Association of α4 with PP2A, PP4, and PP6 has been linked to rapamycin sensitivity [30]–[32], but this is controversial [34]. In particular, some cellular effects of rapamycin have been described to be independent of mTOR kinase activity [48], [49]. For instance, rapamycin inhibits differentiation of C2C12 cells, which is independent of mTOR kinase activity [48], despite of existence of disputation [50]. This promoted us to determine whether rapamycin activation of PP2A is through inhibition of mTOR kinase activity. Here we demonstrated that rapamycin failed to activate PP2A in the cells expressing rapamycin-resistant mTOR (mTORrr), but not in control cells expressing the empty vector (pcDNA3) (Fig. 1), suggesting that rapamycin activation of PP2A is indeed through inhibiting mTOR activity. This is further supported by the observations that 1) rapamycin inhibited IGF-1 stimulated phosphorylation of Erk1/2 in the control cells, but not in the cells expressing mTORrr (Fig. 2B,C); 2) downregulation of mTOR, mimicking the effect of rapamycin, activated PP2A (Fig. 1D) and inhibited IGF-1 stimulated phosphorylation of Erk1/2 (Fig. 2D). The present data are consistent with our previous results that the kinase activity of mTOR is necessary for IGF-1 stimulated F-actin reorganization and cell motility [23], [24].

We found that PD98059 (10 µM) alone did not significantly affect the basal mobility of cells, but dramatically inhibited IGF-1-induced cell motility (Fig. 5). However, when used in combination with rapamycin, PD98059 did not show a significant additive or synergistic effect on rapamycin inhibition of IGF-1-stimulated cell motility (Fig. 5). By contrast, inhibition of PP2A with OA (100 nM) or expression of dn-PP2A significantly attenuated the inhibitory effect of rapamycin on IGF-1-stimulated cell motility (Fig. 3, 4). However, we also noticed that PD98059 was able to block IGF-1 stimulated motility of the cells expressing dn-PP2A (Fig. 4C). The data imply that PD98059 and rapamycin may redundantly inhibit Erk1/2. PD98059 inhibits Erk1/2 by directly inhibiting MEK1/2, whereas rapamycin inactivates Erk1/2 by direct inhibition of mTOR, causing activation of PP2A. In addition, the results also indicate that the basal and the IGF-1-induced motility of cells may be regulated through different mechanisms. Though the basal or IGF-1-induced cell motility is sensitive to rapamycin, only the latter appears to be Erk1/2-dependent. Possibly, the basal motility of cells is associated with considerable activation of S6K1 and/or phosphorylation of 4E-BP1 [23]. It appears that mTOR-mediated cell motility requires a coordination of multiple signaling molecules, at least including Erk1/2, S6K1 and 4E-BP1. Our data suggest that full phosphorylation of Erk1/2, S6K1 and 4E-BP1 is essential for a maximal cell motility.

Here we show that rapamycin inhibited IGF-1 stimulated phosphorylation of Erk1/2, which is related to activation of PP2A. This is consistent with recent findings that rapamycin inhibited IGF-1 or EGF stimulated phosphorylation of Erk1/2, not through inhibiting the upstream kinase, as rapamycin did not inhibit growth factor-induced phosphorylation of MKK1 (Ser217/221) [46]. Instead, both IGF-1 and EGF caused dissociation of PP2Ac from Erk1/2, which was partially prevented by rapamycin [46]. Downregulation of raptor inhibits IGF-1 stimulated phosphorylation of Erk1/2 [46] suggests that mTORC1 participates in the regulation of PP2A-Erk1/2 pathway.

It has been described that IGF-1 activates Erk1/2 by dissociating from Shc from PP2Ac [51]. To determine whether mTORC1 regulates PP2A-Erk1/2 interaction by affecting the association of PP2Ac with Shc, serum-starved Rh1 or Rh30 cells were pretreated with or without rapamycin (100 ng/ml) for 2 h, and then stimulated with or without IGF-1 (10 ng/ml) for 15 min, followed by co-immunoprecipitation (with antibodies to either Shc or PP2Ac). We found that neither IGF-1 (10 ng/ml, 15 min) nor rapamycin (100 ng/ml, 2 h) affected the association of PP2Ac with Shc in the cells (data not shown). The results are, to some extent, in contrast to the previous findings in HIRc B cells (Rat-1 fibroblasts overexpressing human insulin receptors), in which PP2Ac associated with Shc at the basal state and dissociated from it in response to IGF-1 stimulation [51]. This is probably due to cell type context, which has been reported by Yumoto et al. [52]. Clearly, more studies are required to address the mechanism by which mTORC1 regulates PP2A-Erk1/2 signaling.

In the studies, we also assessed the roles of other PP2A family members, such as PP4, PP6 and PP5, in mTOR-mediated cell motility, since these Ser/Thr protein phosphatses have been reported as novel downstream molecules of mTOR as well [30]–[36], [53], [54]. We found that downregulation of PP4 or PP6 neither altered the basal or IGF-1 stimulated cell motility, nor affected rapamycin inhibition of the cell motility in Rh1 and Rh30 cells. However, downregulation of PP5 reduced the basal or IGF-1 stimulated cell motility by ∼50%, and slightly enhanced rapamycin inhibition of cell motility in Rh1 or Rh30 cells. The data suggest that PP5 is a positive regulator for cell motility, and PP4 and PP6 may not be involved in the regulation of cell motility. It appeared that PP5 regulates cell motility in an Erk1/2-independent manner, because downregulation of PP5 did not obviously affect the basal or IGF-1 stimulated phosphorylation of Erk1/2. How PP5 regulates cell motility is under investigation.

In summary, here we have shown that rapamycin inhibits IGF-1 stimulated cell motility by activating PP2A and concurrently inhibiting phosphorylation of Erk1/2. Pharmacological or genetic inhibition of PP2A attenuated the inhibitory effect of rapamycin on IGF-1-stimulated phosphorylation of Erk1/2 as well as cell motility, whereas expression of constitutively active MKK1 results in robust phosphorylation of Erk1/2 and confers resistance to rapamycin inhibition of IGF-1 stimulated cell motility. The results suggest that rapamycin inhibits cell motility, in part by targeting PP2A-Erk1/2 pathway.
